# Spondylodiscitis and an aortic aneurysm due to *Campylobacter coli*

**DOI:** 10.1186/1476-0711-9-8

**Published:** 2010-02-05

**Authors:** Xavier Lemaire, Caroline Dehecq, Christian Cattoen, Laurence Destrieux Garnier, Béatrice Sarraz Bournet, Yazdan Yazdanpanah, Eric Senneville

**Affiliations:** 1 Department of Infectious Diseases, CH Dron Tourcoing, France; 2 Department of Microbiology, CH Valenciennes, Valenciennes, France; 3 Department of Cardio-Vascular Surgery, Hopital cardiologique, Lille, France; 4 Department of Cardio-Vascular Surgery, CH Dron, Tourcoing, France

## Abstract

*Campylobacter coli *is a rare cause of bacteremia. We report here the first case of *C.coli *spondylodiscitis complicated by an aortic aneurysm. Outcome was favourable with surgery and antibiotic therapy.

## Background

While *Campylobacter fetus *is a rare but well-recognized cause of bacteremia [1-3], only a few cases of *Campylobacter coli *bacteremia have been reported in the literature. To the best of our knowledge, no aortic aneurysm due to *C.coli *has been previously described. We report here the first case of *C.coli *spondylodiscitis complicated by an aortic aneurysm.

## Case Presentation

**Figure 1:**
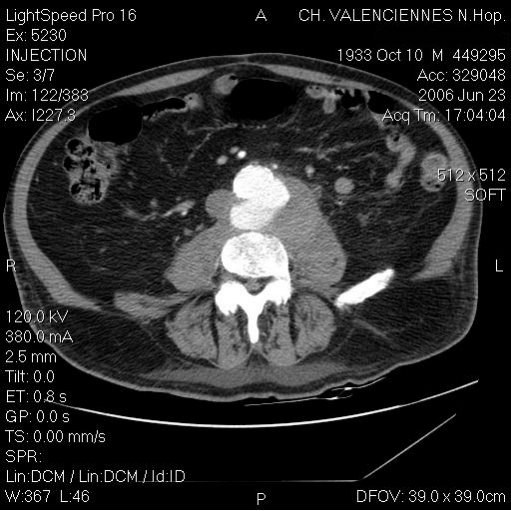
**An axial CT image (the first to be performed for this patient, at the General Hospital, Valenciennes-France) with contrast of the lumbar area showing abdominal aortic aneurysm**.

**Figure 2:**
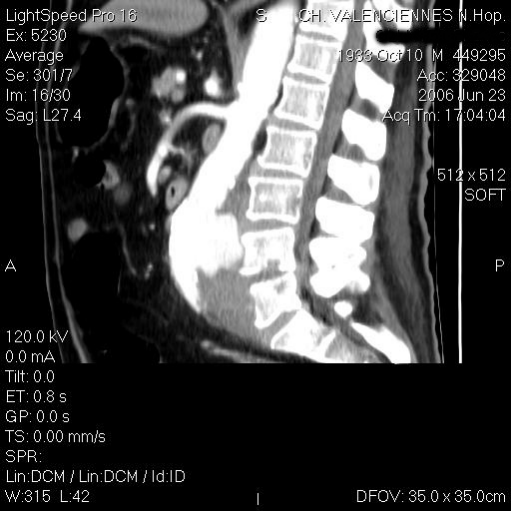
**A sagittal CT image of the lumbar area with contrast reconstruction showing abdominal aortic aneurysm**.

A 72-year old man was admitted in June 2006 in the Rheumatology Department of the General Hospital of Valenciennes-France, for a history of lumbar pain evolving for one month. He had a previous medical history of myocardial injury, dyslipidaemia and tobacco intoxication; he had neither diabetes mellitus nor immunosuppressive therapy. The patient lived in a city and had only a dog. No chills or rigors, but several episodes of nocturnal fever, were noted. At admission, the patient was afebrile, and reported only lumbar back pain and sciatic pain. White blood cell count was 13,000 G/L, ESR 89 mm/h and CRP 138.5 mg/L. Blood cultures taken at admission grew a Gram-negative bacillus. The strain was identified as *C.coli *with the API Campy identification system, and was susceptible to all tested antibiotics. Treatment with a combination of cefotaxime plus gentamicin was immediately started. MRI showed abnormalities consistent with a L4-L5 spondylodiscitis associated with prevertebral and left psoas abscesses. Abdominal CT scan showed a ruptured abdominal aortic aneurysm and a contiguous left psoas collection (figure 1, 2). The patient was transferred to the University Hospital of Lille to be operated on for insertion of endovascular prosthesis. He was then transferred to our Infectious Disease Unit where antibiotic treatment for the aneurysm and spondylodiscitis was continued.

In our unit, the patient remained afebrile without pain. Nevertheless, C-reactive protein continued to markedly rise to 105 mg/L. Ciprofloxacin was added to cefotaxime, and gentamicin was stopped after two weeks of treatment. The day after his admission to our unit, septic shock occurred. CT scan showed the persistence of a 10 × 7.7 × 3.7 cm peri-aortical collection, maybe due to a hematoma related to the first surgical intervention. Surgical drainage was immediately performed (cultures from intraoperative samples were sterile). Postoperative evolution was favourable except for slight resolvable renal failure. CT scan carried out two weeks after surgery showed a marked reduction in the size of the collection and a decrease in the CRP level to 16 mg/L at the same time. Transoesophagal echocardiography was normal. A coloscopy was performed which revealed only a 5 mm non-resecable caecal polyp. Cefotaxime was stopped after two weeks and ciprofloxacin was switched to oral form and is still ongoing. 4 months after surgery, the CT scan showed the disappearance of the initial collection. Two years later, the patient continues to be in good health and remains totally asymptomatic.

## Discussion

Except for one study [4], the incidence of *C.coli *bacteremia remains unknown [5] but seems to be very rare. This is likely due to the susceptibility of this pathogen to the antibacterial activity of the serum [6]. This could explain why *C.coli *bacteremia episodes are often reported in immunocompromised patients or in children [5,7-12]. Only a few studies report bacteremia occurring in immunocompetent patients during acute enteritis [4,13]. Therefore, the incidence of *C.coli *bacteremia may be underestimated due to infrequent blood sampling at the early stage of infection, and inappropriate culture conditions [14].

Although bacteremia may be misdiagnosed, aortic aneurysm and spondylodiscitis are more highly visible infections. Nevertheless, no cases of these localizations have been reported for *C.coli*. We found only one case of meningitis due to this pathogen, but observed no arthritis, endocarditis, pneumonia or prostatitis. We have no evidence for an associated endocarditis and colonoscopy revealed only a small non-inflammatory polyp. We also did not find any cause of immunosuppression in our patient except for age >65 years. Skirrow et al [4] found the average incidence of *Campylobacter *bacteremia to be 1.5/1,000 intestinal *Campylobacter *infections in their total population, and 5.9/1000 in patients aged 65 or over. A combination of cephalosporin and gentamicin was chosen in an attempt to obtain a synergistic bactericidal effect during the first ten days of treatment. Ciprofloxacin was prescribed as prolonged antibiotic therapy in an attempt to obtain permanent high concentrations at the infected site. Although a 3 months duration of treatment for abdominal aortic aneurysms infected with Campylobacter has been evocated in two cases [15,16], considering that an endovascular prosthesis had been inserted in an infected site and the risk of relapsing infection, we considered a life-long antibiotic suppressive therapy as reported for other bacteria [17,18]. A prolonged course of ciprofloxacin was then proposed to the patient due to its activity toward foreign body infection due to Gram-negative bacilli, as demonstrated in both experimental and clinical studies [19-22].

Concerning the spondylodiscitis, despite some reported studies [23-25], as drainage of the prevertebral and left psoas abscesses was done during the first surgical intervention; no specific intervention was then performed.

We believe that the spondylodiscitis was primarily due to *C.coli *bacteremia, but it is unclear as to whether the aneurysm rupture was secondary to bacteremia or to erosion of the arterial wall by the abscess.

A limitation of our case report is that species identification was made with the API Campy identification system and not by PCR. As previously reported the sensibility of the API Campy test, particularly for identification of *C.coli*, is poor [26-28]. Despite imperfect specificity of this test, diagnosis of *C.coli *was always confirmed by PCR in these studies. In accordance with these data, the possibility of misdiagnosing *Campylobacter *species in the present case seems to be very low.

## Conclusion

Although it had not yet been described, *C.coli *may be responsible for spondylodiscitis complicating bacteremia. Like *C.fetus*, *C.coli *may, under conditions not yet elucidated, cross the intestinal barrier and cause severe metastatic infections.

## Consent

Written informed consent was obtained from the patient for publication of this case report and any accompanying images. A copy of the written consent is available for review by the Editor-in-Chief of this journal.

## Competing interests

The authors declare that they have no competing interests.

## Authors' contributions

XL have made substantial contributions to acquisition of data, have been involved in drafting the manuscript. CD have been involved in drafting the manuscript. CC have made substantial contributions to acquisition of data. LDG have made substantial contributions to acquisition of data. BSB have made substantial contributions to acquisition of data. YY have given final approval of the version to be published. ES have made substantial contributions to conception of the case redaction, have given final approval of the version to be published. All authors read and approved the final manuscript.
